# No association between alcohol consumption and pancreatic cancer even among individuals genetically susceptible to the carcinogenicity of alcohol

**DOI:** 10.1038/s41598-021-94111-w

**Published:** 2021-07-15

**Authors:** Yan-Shen Shan, Li-Tzong Chen, Chih-Hsing Wu, Yin-Fan Chang, Chih-Ting Lee, Nai-Jung Chiang, Ying-Jui Chao, Chia-Jui Yen, Hui-Jen Tsai, Hsin-En Huang, Chia-Rung Tsai, Ya-Ling Weng, Han-Chien Yang, Hui-Chin Liu, Jeffrey S. Chang

**Affiliations:** 1 Department of Surgery, National Cheng Kung University Hospital, College of Medicine, National Cheng Kung University, 138 Sheng Li Road, Tainan, 70456 Taiwan; 2 Institute of Clinical Medicine, College of Medicine, National Cheng Kung University, Tainan, 138 Sheng Li Road, Tainan, 70456 Taiwan; 3 National Institute of Cancer Research, National Health Research Institutes, 1F No 367, Sheng-Li Road, Tainan, 70456 Taiwan; 4 Department of Internal Medicine, National Cheng Kung University Hospital, College of Medicine, National Cheng Kung University, 138 Sheng Li Road, Tainan, 70456 Taiwan; 5 Department of Internal Medicine, Kaohsiung Medical University Hospital, Kaohsiung Medical University, Ziyou 1st Road, Sanmin District, Kaohsiung, 80756 Taiwan; 6 Institute of Molecular Medicine, College of Medicine, National Cheng Kung University, 138 Sheng Li Road, Tainan, 70456 Taiwan; 7 Department of Family MedicineNational Cheng Kung University Hospital, College of Medicine, National Cheng Kung University, 138 Sheng Li Road, Tainan, 70456 Taiwan; 8 Institute of Geriatrics, College of Medicine, National Cheng Kung University, 1 University Road, Tainan, 701 Taiwan; 9 Department of Family Medicine, Mennonite Christian Hospital, 44 Min Chuan Road, Hualien, 970 Taiwan; 10 Department of Nursing, National Cheng Kung University HospitalCollege of Medicine, National Cheng Kung University, 138 Sheng Li Road, Tainan, 70456 Taiwan

**Keywords:** Oncology, Risk factors

## Abstract

Inconsistent results have been reported for the association between alcohol use and pancreatic cancer, particularly at low levels of alcohol consumption. Individuals genetically susceptible to the carcinogenic effect of alcohol might have higher pancreatic cancer risk after drinking alcohol. The current study investigated the association between alcohol use and pancreatic cancer with 419 pancreatic cancer cases and 963 controls recruited by a hospital-based case–control study in Taiwan. Gene-environment interaction between alcohol use and polymorphisms of two ethanol-metabolizing genes, *ADH1B* and *ALDH2*, on pancreatic risk was evaluated. Our results showed no significant association between alcohol drinking and an increased pancreatic cancer risk, even at high levels of alcohol consumption. Even among those genetically susceptible to the carcinogenic effect of alcohol (carriers of ADH1B*2/*2(fast activity) combined with ALDH2*1/*2(slow activity) or ALDH2*2/*2(almost non-functional)), no significant association between alcohol use and pancreatic cancer was observed. Overall, our results suggested that alcohol drinking is not a significant contributor to the occurrence of pancreatic cancer in Taiwan.

## Introduction

Pancreatic cancer is a highly lethal cancer with a very low survival rate (5-year survival rate = 9%)^1^. During 1990 to 2017, the worldwide incidence of pancreatic cancer increased from 5.0 per 100,000 person-years to 5.7 per 100,000 person years, with approximately 448,000 incident cases occurring in 2017^2^. Cigarette smoking, diabetes, and obesity are the three established risk factors of pancreatic cancer. The percentages of global pancreatic deaths attributed to smoking, diabetes, and obesity were estimated to be 25.9%, 9.3%, and 5.0%, respectively for men, and 16.1%, 8.6%, and 7.4%, respectively, for women^2^. Other factors showing strong positive associations with pancreatic cancer include no history of allergies^3^, poor oral hygiene/health^4^, and diet with lower amounts of fruits and vegetables^5–7^.

Many studies have investigated the association between alcohol consumption and pancreatic cancer and the results have been inconsistent, particularly at low levels of alcohol consumption. More consistent results were found for the association between heavy alcohol consumption and pancreatic cancer^8–10^. Although studies generally did not support a positive association between low to moderate levels of alcohol consumption and pancreatic cancer, East Asians may be more susceptible to the carcinogenic effect of alcohol due to a highly prevalent single nucleotide polymorphism (SNP), *ALDH2* rs671. ALDH2, an enzyme encoded by the *ALDH2* gene, is responsible for converting acetaldehyde, a carcinogenic product of ethanol metabolism, to acetate. Rs671 has two alleles, with codon 504 showing a glutamate for *1 and a lysine for *2^11^. The replacement of glutamate (*1) with lysine (*2) reduces the enzyme activity dramatically with ALDH2*2/*2 generating an enzyme with only 4% activity and the heterozygous ALDH2*1/*2 producing an enzyme with < 50% activity^12^. The ALDH2*2 is rare (prevalence < 5%) among the Europeans and Africans^13^, but very common among East Asians (Chinese, Japanese, Korean, and Taiwanese), with 30–50% of individuals carrying at least a single copy of the *2 allele^14^. Besides *ALDH2*, *ADH1B* is another gene that affects the metabolism of ethanol by generating an enzyme that converts ethanol into acetaldehyde. Codons 48 (Arg48His; rs1229984) and 370 (Arg370Cys; rs2066702) together determine the three alleles of *ADH1B*. Arginine at codon 48 (Arg48) + arginine at codon 370 (Arg370) = ADH1B *1 allele, histidine at codon 48 (His48) + Arg370 = ADH1B*2 allele, and Arg48 + cysteine at codon 370 (Cys370) = ADH1B*3 allele^15^. Approximately 60–85% Pacific Rim Asians carry the ADH1B*2 allele^16^ and the enzyme encoded by the ADH1B*2/*2 genotype metabolizes ethanol to acetaldehyde 40 times faster than the enzyme generated by the ADH1B*1/*1 genotype according to in vitro finding^17^. The ADH1B*3 allele is found mainly among individuals of African descent and the enzyme encoded by the *3 allele has 15% higher activity than the one encoded by the *1 allele^18^.

Previously, a case–control study by Kanda et al. from Japan with 160 cases and 1,600 controls showed that the highest pancreatic cancer risk was observed among ever drinkers who carry the ADH1B*2/*2(fast activity) in combination with ALDH2*1/*2(slow activity) or ALDH2*2/*2(almost non-functional)^19^. Individuals with these genotype combinations can generate acetaldehyde rapidly after drinking alcohol but are slow at metabolizing acetaldehyde to non-carcinogenic acetate and may thus have a higher susceptibility to the carcinogenic effect of alcohol. Because the study by Kanda et al. has been the only study examining the combined effect of *ADH1B* and *ALDH2* polymorphisms on the association between alcohol drinking and pancreatic cancer with a relatively small case sample size (n = 160), the current study aimed to investigate this topic with 419 pancreatic cancer cases and 963 controls recruited from a hospital in Taiwan, where the prevalence of ALDH2*2 carriers is the highest in the world (~ 50%).

## Results

The current analysis included 419 pancreatic cancer cases and 963 controls with a participation rate of 80% for the cases and 81% for the controls. Ninety percent of the cases were interviewed within 6 months of the pancreatic cancer diagnosis (82% within 3 months and 8% between 3–6 months). The study used frequency matching by age and sex for control selection; however, because of the ongoing recruitment of study subjects, the frequency matching process has not been completed, resulting in the imbalanced distributions of age and sex between the cases and the controls. Compared to the controls, cases had an older average age, a lower percentage of women, and a lower percentage of individuals with at least a college education (Table 1).

**Table 1:** Demographic characteristics of the pancreatic cancer patients and control subjects.

Characteristics	Cases N = 419 n (%)	Controls N = 963 n (%)	P-value^a^
**Age (years)**			
Mean (SE)	63.4 (0.5)	58.3 (0.4)	< 0.0001
**Sex**			
Men	237 (56.6)	408 (42.4)	< 0.0001
Women	182 (43.4)	555 (57.6)	
**Education**			
≦ Junior high	199 (47.5)	259 (26.9)	< 0.0001
High school/technical school	119 (28.4)	252 (26.1)	
College	86 (20.5)	354 (36.8)	
Graduate school	15 (3.6)	98 (10.2)	

*N* number, *SE* standard error.

^a^P-values were generated using T-tests (for continuous variables) or chi-squared tests (for categorical variables).

Supplementary 1 presents the associations between the potential confounding factors and pancreatic cancer. Cigarette smoking, poor oral hygiene, diabetes/glucose intolerance, and obesity (BMI ≧ 27 kg/m^2^) were all associated with an increased pancreatic cancer risk. More frequent consumption of vegetables and history of allergy were associated with a reduced pancreatic cancer. These factors were all adjusted as confounders in the subsequent analyses for the association between alcohol use and pancreatic cancer.

No significant association was observed between pancreatic cancer and the status, frequency, or level of alcohol drinking (Table 2). In addition, the association between alcohol use and pancreatic cancer did not differ significantly by the cigarette smoking status (Table 3).

**Table 2:** The association between alcohol drinking and pancreatic cancer risk.

Alcohol consumption	Cases N = 419 n (%)	Controls N = 963 n (%)	OR (95% CI)^a^
**Drinking status**			
Never	152 (36.3)	329 (34.2)	Reference
Occasional	172 (41.0)	486 (50.5)	0.82 (0.60–1.11)
Former regular	34 (8.1)	60 (6.2)	0.74 (0.42–1.31)
Current regular	61 (14.6)	88 (9.1)	1.03 (0.63–1.68)
**Frequency**			
Never	152 (36.3)	329 (34.2)	Reference
Occasional	172 (41.0)	486 (50.5)	0.82 (0.60–1.11)
Monthly	6 (1.4)	13 (1.3)	0.94 (0.31–2.84)
Weekly	21 (5.0)	53 (5.5)	0.57 (0.29–1.12)
Daily	55 (13.1)	74 (7.7)	0.94 (0.56–1.57)
Unknown	13 (3.1)	13 (0.8)	–
**Level**			
Never	152 (36.3)	329 (34.2)	Reference
Occasional	172 (41.0)	486 (50.5)	0.82 (0.60–1.11)
Light	44 (10.5)	93 (9.7)	0.67 (0.40–1.13)
Moderate	6 (1.4)	16 (1.7)	0.46 (0.15–1.39)
Heavy	24 (5.7)	26 (2.7)	1.17 (0.57–2.38)
Unknown	21 (5.0)	13 (1.4)	–

*CI* confidence interval, *N* number, *OR* odds ratio.

^a^OR and 95% CI were calculated using unconditional logistic regression, adjusted for sex, age, education, cigarette smoking (pack-years), oral hygiene score, vegetable consumption, allergy, diabetes/glucose intolerance and BMI at two years before the pancreatic cancer diagnosis for the cases or before the interview date for the controls.

**Table 3:** The association between alcohol drinking and pancreatic cancer risk stratified by cigarette smoking.

Alcohol consumption	Cases n (%)	Controls n (%)	OR (95% CI)^a^	Cases n (%)	Controls n (%)	OR (95% CI)^a^
	Never smokers			Ever smokers		
**Drinking Status**						
Never	122 (47.8)	299 (40.8)	Reference	30 (18.3)	30 (13.8)	Reference
Occasional	113 (44.3)	397 (53.2)	0.83 (0.59–1.17)	59 (36.0)	89 (41.0)	0.79 (0.38–1.63)
Former regular	7 (2.8)	16 (2.1)	1.23 (0.45–3.41)	27 (16.5)	44 (20.3)	0.65 (0.28–1.50)
Current regular	13 (5.1)	34 (4.6)	1.00 (0.44–2.23)	48 (29.3)	54 (24.9)	0.98 (0.46–2.12)
	*P*-interaction = 0.74					
**Frequency**						
Never	122 (47.8)	299 (40.8)	Reference	30 (18.3)	30 (13.8)	Reference
Occasional	113 (44.3)	397 (53.2)	0.83 (0.59–1.17)	59 (36.0)	89 (41.0)	0.79 (0.38–1.64)
Monthly	5 (2.0)	8 (1.1)	2.08 (0.59–7.34)	1 (0.6)	5 (2.3)	0.16 (0.02–1.66)
Weekly	3 (1.2)	18 (2.4)	0.49 (0.13–1.89)	18 (11.0)	35 (16.1)	0.60 (0.25–1.49)
Daily	7 (2.8)	20 (2.7)	0.89 (0.31–2.60)	48 (29.3)	54 (24.9)	0.94 (0.43–2.03)
Unknown	5 (2.0)	4 (0.5)	–	8 (4.9)	4 (1.8)	–
	*P*-interaction = 0.34					
**Level**						
Never	122 (47.8)	299 (40.8)	Reference	30 (18.3)	30 (13.8)	Reference
Occasional	113 (44.3)	397 (53.2)	0.83 (0.59–1.17)	59 (36.0)	89 (41.0)	0.77 (0.37–1.61)
Light	10 (3.9)	32 (4.3)	0.89 (0.37–2.13)	34 (20.7)	61 (28.1)	0.56 (0.25–1.25)
Moderate	1 (0.4)	5 (0.7)	0.82 (0.09–7.95)	5 (3.0)	11 (5.1)	0.38 (0.10–1.49)
Heavy	2 (0.8)	7 (0.9)	0.75 (0.13–4.20)	22 (13.4)	19 (8.8)	1.36 (0.53–3.51)
Unknown	7 (2.8)	6 (0.8)	–	14 (8.5)	7 (3.2)	–
	*P*-interaction = 0.90					

*CI* confidence interval, *N* number; *OR* odds ratio.

a. OR and 95% CI were calculated using unconditional logistic regression, adjusted for sex, age, education, cigarette smoking (pack-years), oral hygiene score, vegetable consumption, allergy, diabetes/glucose intolerance and BMI at two years before the pancreatic cancer diagnosis for the cases or before the interview date for the controls.

The polymorphisms of *ADH1B* and *ALDH2* showed no significant associations with pancreatic cancer risk (Table 4). No significant association between alcohol use and pancreatic cancer was observed after stratification by the genotypes of *ADH1B* or *ALDH2* (Table 5) or by the combinations of *ADH1B* and *ALDH2* genotypes (Table 6).

**Table 4:** The association between *ALDH2* rs671, and *ADH1B* rs1229984 and pancreatic cancer risk.

	**Cases** **n (%)**	**Controls** **n (%)**	OR (95% CI)^a^
***ADH1B* rs1229984**			
TT (*2/*2)( Fast)	208 (51.9)	502 (54.6)	Referent
CT (*1/*2)	156 (38.9)	334 (36.4)	1.11 (0.84–1.48)
CC (*1/*1)(Slow)	37 (9.2)	83 (9.0)	1.20 (0.74–1.94)
***ALDH2* rs671**			
GG (*1/*1)(Normal)	193 (47.2)	450 (47.9)	Referent
AG (*1/*2)	187 (45.7)	407 (43.3)	1.18 (0.90–1.56)
AA (*2/*2) (Non-functional)	29 (7.2)	83 (8.8)	0.75 (0.44–1.25)
***ADH1B* rs1229984 + *ALDH2* rs671**			
Group 1: Fast *ADH1B* (*2/*2) + normal *ALDH2* (*1/*1)	101 (25.1)	233 (25.6)	Referent
Group 2: Fast *ADH1B* (*2/*2) + slow/non-functional *ALDH2* (*1/*2 + *2/*2)	107 (26.7)	266 (29.2)	0.98 (0.68–1.42)
Group 3: Slow *ADH1B* (*1/*1 + *1/*2) + normal *ALDH2* (*1/*1)	89 (22.2)	203 (22.3)	1.00 (0.67–1.48)
Group 4: Slow *ADH1B* (*1/*1 + *1/*2) + slow/non-functional *ALDH2* (*1/*2 + *2/*2)	104 (25.9)	210 (23.0)	1.23 (0.84–1.79)

*CI* confidence interval, *N* number, *OR* odds ratio.

^a^OR and 95% CI were calculated using unconditional logistic regression, adjusted for sex, age, education, cigarette smoking (pack-years), oral hygiene score, vegetable consumption, allergy, diabetes/glucose intolerance and BMI at two years before the pancreatic cancer diagnosis for the cases or before the interview date for the controls.

**Table 5:** The association between alcohol drinking and pancreatic cancer risk stratified by the genotypes of *ADH1B* or *ALDH2.*

	**Cases** **n (%)**	**Controls** **n (%)**	**OR (95% CI)**^a^	**Cases** **n (%)**	**Controls** **n (%)**	**OR (95% CI)**^a^
***ADH1B* rs1229984**	Fast *ADH1B* (*2/*2)			Slow *ADH1B* (*1/*1 + *1/*2)		
**Drinking status**						
Never	81 (38.9)	175 (34.9)	Reference	65 (33.7)	138 (33.1)	Reference
Occasional	79 (38.0)	241 (48.0)	0.74 (0.48–1.14)	84 (43.5)	227 (54.4)	0.92 (0.57–1.47)
Former regular	19 (9.1)	38 (7.6)	0.70 (0.33–1.49)	13 (6.7)	17 (4.1)	0.78 (0.29–2.12)
Current regular	29 (13.9)	48 (9.6)	0.94 (0.47–1.88)	31 (16.1)	35 (8.4)	1.35 (0.63–2.89)
	*P*-interaction = 0.39					
**Frequency**						
Never	81 (38.9)	175 (34.9)	Reference	65 (33.7)	138 (33.1)	Reference
Occasional	79 (38.0)	241 (48.0)	0.73 (0.47–1.13)	84 (43.5)	227 (54.4)	0.92 (0.57–1.47)
Monthly	3 (1.4)	5 (1.0)	1.55 (0.30–7.92)	3 (1.6)	7 (1.7)	0.63 (0.12–3.18)
Weekly	10 (4.8)	31 (6.2)	0.40 (0.16–1.02)	11 (5.7)	18 (4.3)	1.12 (0.38–3.33)
Daily	29 (13.9)	45 (9.0)	0.87 (0.43–1.77)	23 (11.9)	24 (5.8)	1.10 (0.47–2.61)
Unknown	6 (2.9)	5 (1.0)	–	7 (3.6)	3 (0.7)	–
	*P*-interaction = 0.42					
**Level**						
Never	81 (38.9)	175 (34.9)	Reference	65 (33.7)	138 (33.1)	Reference
Occasional	79 (38.0)	241 (48.0)	0.73 (0.48–1.14)	84 (43.5)	227 (54.4)	0.92 (0.57–1.47)
Light	25 (12.0)	51 (10.2)	0.70 (0.33–1.44)	17 (8.8)	38 (9.1)	0.56 (0.24–1.34)
Moderate	2 (1.0)	8 (1.6)	0.20 (0.03–1.23)	4 (2.1)	5 (1.2)	1.74 (0.38–8.00)
Heavy	10 (4.8)	18 (3.6)	0.80 (0.31–2.10)	13 (6.7)	6 (1.4)	2.34 (0.65–8.49)
Unknown	11 (5.3)	9 (1.8)	–	10 (5.2)	3 (0.7)	–
	*P*-interaction = 0.15					
***ALDH2* rs671**	Normal *ALDH2* (*1/*1)			Slow/non-functional *ALDH2* (*1/*2 + *2/*2)		
**Drinking status**						
Never	54 (28.0)	127 (28.2)	Reference	96 (44.4)	191 (39.0)	Reference
Occasional	84 (43.5)	238 (52.9)	0.97 (0.59–1.57)	81 (37.5)	239 (48.8)	0.66 (0.44–1.01)
Former regular	16 (8.3)	30 (6.7)	0.80 (0.33–1.92)	18 (8.3)	29 (5.9)	0.66 (0.30–1.48)
Current regular	39 (20.2)	55 (12.2)	1.11 (0.53–2.32)	21 (9.7)	31 (6.3)	1.11 (0.52–2.33)
	*P*-interaction = 0.61					
**Frequency**						
Never	54 (28.0)	127 (28.2)	Reference	96 (44.4)	191 (39.0)	Reference
Occasional	84 (43.5)	238 (52.9)	0.96 (0.59–1.56)	81 (37.5)	239 (48.8)	0.66 (0.44–1.01)
Monthly	3 (1.5)	8 (1.8)	0.96 (0.20–4.69)	3 (1.4)	5 (1.0)	0.98 (0.19–5.11)
Weekly	12 (6.2)	28 (6.2)	0.54 (0.20–1.46)	9 (4.2)	23 (4.7)	0.63 (0.24–1.66)
Daily	31 (16.1)	46 (10.2)	0.91 (0.41–2.03)	23 (10.6)	27 (5.5)	1.04 (0.48–2.23)
Unknown	9 (4.7)	3 (0.7)	–	4 (1.8)	5 (1.0)	–
	*P*-interaction = 0.63					
**Level**						
Never	54 (28.0)	127 (28.2)	Reference	96 (44.4)	191 (39.0)	Reference
Occasional	84 (43.5)	238 (52.9)	0.95 (0.58–1.55)	81 (37.5)	239 (48.8)	0.66 (0.44–1.01)
Light	25 (13.0)	56 (12.4)	0.58 (0.26–1.30)	18 (8.3)	35 (7.1)	0.78 (0.36–1.69)
Moderate	4 (2.1)	8 (1.8)	0.63 (0.15–2.66)	2 (0.9)	7 (1.4)	0.34 (0.05–2.44)
Heavy	14 (7.3)	16 (3.6)	1.33 (0.49–3.61)	10 (4.6)	10 (2.0)	1.13 (0.39–3.29)
Unknown	12 (6.2)	5 (1.1)	–	9 (4.2)	8 (1.6)	–
	*P*-interaction = 0.61					

*CI* confidence interval, *N* number, *OR* odds ratio.

^a^OR and 95% CI were calculated using unconditional logistic regression, adjusted for sex, age, education, cigarette smoking (pack-years), oral hygiene score, vegetable consumption, allergy, diabetes/glucose intolerance and BMI at two years before the pancreatic cancer diagnosis for the cases or before the interview date for the controls.

**Table 6:** The association between alcohol drinking and pancreatic cancer risk by the combination of *ALDH2* rs671 and *ADH1B* rs1229984.

Ethanol metabolism group								
**Alcohol consumption**^**a**^	**Group 1** Fast *ADH1B* (*2/*2) + Normal *ALDH2* (*1/*1)		**Group 2** Fast *ADH1B* (*2/*2) Slow/non-functional *ALDH2* (*1/*2 + *2/*2)		**Group 3** Slow *ADH1B* (*1/*1 + *1/*2) Normal *ALDH2* (*1/*1)		**Group 4** Slow *ADH1B* (*1/*1 + *1/*2) Slow/non-functional *ALDH2* (*1/*2 + *2/*2)	
	Ca/Co	OR (95% CI)^a^	Ca/Co	OR (95% CI)^a^	Ca/Co	OR (95% CI)^a^	Ca/Co	OR (95% CI)^a^
**All head and neck cancer**								
Never	32/72	Referent	49/101	Referent	22/51	Referent	43/84	Referent
Occasional	44/107	0.97 (0.49–1.93)	35/133	0.46 (0.24–0.86)	39/123	0.83 (0.38–1.84)	45/103	1.11 (0.58–2.12)
Former regular	8/22	0.44 (0.13–1.51)	11/16	0.89 (0.30–2.60)	6/8	0.98 (0.20–4.74)	7/9	0.73 (0.18–2.98)
Current regular	17/32	0.78 (0.27–2.31)	12/16	1.18 (0.42–3.34)	22/21	1.49 (0.47–4.67)	9/14	1.37 (0.42–4.50)
	*P*-interaction = 0.20							
Never	32/72	Referent	49/101	Referent	22/51	Referent	43/84	Referent
Occasional	44/107	0.96 (0.48–1.91)	35/133	0.46 (0.24–0.86)	39/123	0.83 (0.38–1.85)	45/103	1.11 (0.58–2.12)
Monthly	1/3	0.70 (0.04–11.86)	2/2	2.34 (0.24–22.75)	2/5	0.81 (0.10–6.23)	1/2	0.30 (0.01–6.84)
Weekly	7/18	0.34 (0.09–1.28)	3/13	0.43 (0.10–1.86)	5/9	1.02 (0.16–6.63)	6/9	1.02 (0.24–4.37)
Daily	13/30	0.55 (0.17–1.77)	16/15	1.25 (0.45–3.50)	16/15	1.01 (0.26–3.92)	7/9	1.73 (0.43–6.87)
Unknown	4/3	–	2/2	–	5/0	–	2/3	–
	*P*-interaction = 0.31							
Never	32/72	Referent	49/101	Referent	22/51	Referent	43/84	Referent
Occasional	44/107	0.97 (0.49–1.92)	35/133	0.44 (0.23–0.84)	39/123	0.84 (0.37–1.90)	45/103	1.12 (0.59–2.14)
Light	15/33	0.52 (0.17–1.61)	10/18	0.88 (0.29–2.66)	9/22	0.36 (0.09–1.47)	8/16	0.69 (0.20–2.43)
Moderate	2/5	0.37 (0.04–3.39)	0/3	–	2/2	1.53 (0.17–14.09)	2/3	2.90 (0.35–24.15)
Heavy	4/11	0.43 (0.09–2.11)	6/7	1.22 (0.32–4.65)	9/5	1.86 (0.34–10.31)	4/1	6.90 (0.39–121.16)
Unknown	4/5	–	7/4	–	8/0	–	2/3	–
	*P*-interaction = 0.30							

*CA* case, *CI* confidence interval, *CO* control, *OR* odds ratio.

^a^OR and 95% CI were calculated using unconditional logistic regression, adjusted for sex, age, education, cigarette smoking (pack-years), oral hygiene score, vegetable consumption, allergy, diabetes/glucose intolerance and BMI at two years before the pancreatic cancer diagnosis for the cases or before the interview date for the controls.

## Discussion

Our results did not support the association between alcohol drinking and an increased pancreatic cancer risk, even at high levels of alcohol consumption. Even among those genetically susceptible to the carcinogenic effect of alcohol, no significant association between alcohol use and pancreatic cancer was observed.

Consistent with results from previous studies, our study also showed no association between low levels of alcohol use and pancreatic cancer; however, in contrary to the findings of previous studies, our study found no association between high levels of alcohol use and pancreatic cancer. In a pooled analysis combining data of 21 cohorts (n = 4,211,129) from 19 prospective studies, Wang et al. reported that high level of alcohol consumption (≧ 24 g per day) was associated with an elevated pancreatic cancer risk (relative risk (RR) = 1.15, 95% CI 1.06–1.25), while no significant association was observed for low (< 12 g per day) and moderate (12–23.9 g per day) levels of alcohol consumption^10^. Genkinger et al. also performed a pooled analysis with 14 cohort studies (n = 862,664) and reported a 1.2 times increase in pancreatic cancer risk comparing those that drank ≧ 30 g of alcohol per day to those that drank 0 g (RR = 1.22, 95% CI 1.03–1.45)^8^. Lucenteforte et al. performed a pool analysis with 5,585 cases and 11,827 controls from 10 case–control studies, and showed a 1.6 times increase in pancreatic cancer risk for heavy drinking of ≧ 9 drinks per day (OR = 1.6, 95% CI 1.2–2.2), while light to moderate drinking (≦ 4 drinks/day) showed no significant association with pancreatic cancer^9^. A major reason for the non-significant finding in our study might be due to the low percentages of heavy drinkers (5.7% among cases and 2.7 among controls). Given our sample size of heavy drinkers, we calculated that our study has a power = 0.80 to detect an OR of 2.3, and a low statistical power of 0.07, 0.1, and 0.34 to detect a RR or OR of 1.15, 1.22, 1.6, respectively, reported by the previous studies^8–10^. Nevertheless, the low percentages of heavy alcohol drinkers suggested that heavy alcohol drinking is less likely to contribute the development of pancreatic cancer in our study population.

Only one previous study by Kanda et al. evaluated the influence of *ADH1B* and *ALDH2* polymorphisms on the association between alcohol drinking and pancreatic cancer^19^. Consistent with that study, our results also showed no independent association between the polymorphisms of *ADH1B* and *ALDH2* and pancreatic cancer risk. However, in contrast to their findings showing the strongest positive association between alcohol and pancreatic cancer among individuals with the combination of ADH1B*2/*2(fast activity) and ALDH2*1/*2(slow activity) or ALDH2*2/*2(almost non-functional)^19^, our results found no significant association between alcohol drinking and pancreatic cancer regardless of the genotype combinations of *ADH1B* and *ALDH2*. Although chance finding could not be ruled out to explain the discrepant results between the two studies, a likely reason may still be the low percentage of heavy alcohol users in our study population. In the study by Kanda et al. where higher level of alcohol drinking was defined as ≧ 30 g of alcohol per day, 22.5% of the cases and 15.9% of the controls met this definition, whereas in our study population only 7.2% of the cases and 4.1% of the controls met this definition. Overall, our results suggested that in a study population with low percentage of heavy alcohol users, genetic susceptibility to carcinogenicity of alcohol alone is insufficient to increase pancreatic cancer risk.

This study has several limitations. It is difficult to know whether the cases and controls in a hospital-based case–control study are from the same source population. In particular, the representativeness of the control subjects may be questionable. The percentages of ever alcohol users (occasional + regular) in our study were 80% for men and 55.5% for women, which were a little higher than the percentage of ever drinkers (70% for men and 45.3% for women) recorded by the 2013 national health survey^20^. This could have biased our results on the association between alcohol use and pancreatic cancer towards the null. The 2013 national health survey did not record the level of alcohol use; therefore, we could not access the potential bias for heavy alcohol use in our study. Another limitation is that cases might have ruminated more about alcohol use than the controls, biasing the results away from the null. Finally, due to the differences in alcohol use prevalence and behaviors, our results may not be generalizable to populations in other countries. Our results only suggested that alcohol is not a major contributor to the occurrence of pancreatic cancer in Taiwan.

The major strength of the current study is that it is one of the few studies that assessed the combined impact of *ADH1B* and *ALDH2* polymorphisms on the association between alcohol use and pancreatic cancer. It allowed us to show that even among individuals that are genetically susceptible to the carcinogenic effect of alcohol, alcohol use was not associated with pancreatic cancer risk.

In conclusion, our study did not support an association between alcohol use and pancreatic cancer even after considering the level of alcohol use and the influence of *ADH1B* and *ALDH2* polymorphisms.

## Methods

The contents and the execution of the current study received approval from institutional review boards of the National Cheng Kung University Hospital and the National Health Research Institutes. Every potential study participant was informed about the details and the potential risk of participating in the study. Those who agreed to join the study were asked to provide a signed informed consent. The study was conducted according to the guidelines and the regulations set by the institutional review boards of the National Cheng Kung University Hospital and the National Health Research Institutes.

### Subject recruitment

Recruitment of the study subjects was performed by an ongoing case–control study of pancreatic cancer at the National Cheng Kung University Hospital. Recruitment of the pancreatic cancer cases took place at the Department of General Surgery or the Division of Hemato-Oncology, Department of Internal Medicine. Eligible cases were those with: (1) diagnosis of pancreatic ductal adenocarcinoma; (2) no cancer history prior to the diagnosis of pancreatic cancer; (3) age ≧ 20 years; and (4) the capability to comprehend the contents of the study and give informed consent. Recruitment of the controls was conducted at the Department of Family Medicine. The eligible controls were those who: (1) visited the hospital for vaccination, routine physical examination, or for minor illnesses (e.g. common cold, headache) unrelated to cigarette smoking or metabolic diseases (diabetes, hypertension, hyperlipidemia); (2) had no history of cancer; (3) were aged≧ 20 years; and (4) were able to understand the contents of the study and give informed consent. The current study analyzed data of subjects recruited from November 19, 2013 to January 21, 2020.

### In-person interview

In-person interview was conducted to collect data on alcohol use from each study subject. Each participant was first asked whether they ever drank any alcohol with four possible responses: never, once or twice, yes but only occasional at special events, and yes for regular drinking. Regular alcohol drinkers were further asked the followings: (1) whether they had quit drinking for more than 6 months with positive responders defined as former regular alcohol drinkers; (2) type of alcohol consumed including beer, wine, and liquor; and (3) frequency and the volume of alcohol drinking. Data on potential confounders in the association between alcohol use and pancreatic cancer were also collected. These included: (1) demographic characteristics, including age, sex, and education; (2) lifestyle habits, including use of cigarettes and intake of vegetables; (3) medical history regarding allergy and diabetes/glucose intolerance; (4) habits of oral hygiene, including regular dental visits, frequency of tooth brushing, and use of dental floss; and (5) height and weight two years prior to the diagnosis of pancreatic cancer for the cases or before the date of interview for the controls. We collected data on weight two years prior to the diagnosis of pancreatic cancer for the cases to minimize reverse causation in the association between body mass index (BMI) and pancreatic cancer.

### Collection and processing of DNA samples

To obtain DNA, all subjects were asked to provide blood samples collected in vacutainer tubes containing EDTA (lavender-top) or buccal swab samples obtained by gently brushing the buccal mucosa with FLOQSwabs (Copan Flock Technologies, Brescia, Italy). Buffy coat was separated from the blood by centrifugation before DNA extraction. Commercially available DNA purification kit was used to extract genomic DNA from the buffy coat and the buccal swab samples. DNA samples were kept in a − 80 °C refrigerator until ready for genotyping.

### Genotyping

*ADH1B* rs1229984 and *ALDH2* rs671 were genotyped for each study subject using Taqman-based allelic discrimination method on an Applied Biosystems 7500 Real-Time Polymerase Chain Reaction System (Applied Biosystems, Foster City, CA).

### Statistical analysis

T-test was performed to compare the mean age between cases and the controls. The differences in the distributions of sex and education levels between cases and controls were evaluated using chi-squared test.

The odds ratios (OR) and 95% confidence interval (CI) for estimating the association between alcohol use and pancreatic cancer was generated by unconditional logistic regression analysis, adjusted for age, sex, educational level, cigarette smoking, oral hygiene, vegetable intake, history of allergy, history of diabetes/glucose intolerance, and BMI. Alcohol use was evaluated in several ways: (1) by status, including never, occasional, former regular, and current regular; (2) by frequency, including monthly, weekly, and daily; (3) by level, with light drinking defined as < 1 drink/day (14 g of pure alcohol) for women and < 2 drinks/day for men, moderate drinking defined as 1–3 drinks/day for women and 2–4 drinks/day for men, and heavy drinking as > 3 drinks/day for women and > 4 drinks/day for men. The total grams of pure alcohol were calculated according to the frequency and the volume of alcohol consumption and the types of alcoholic beverage consumed. The grams/day of pure alcohol was first calculated for each beverage type with the formula: total volume per day x alcohol content (5% for beer, 13% for wine, and 40% for liquor) x density of ethanol (0.798 g/ml). The grams/day of alcohol for the different beverage types were then added together to generate the total grams/day of pure alcohol.

The association between alcohol use and pancreatic cancer was further stratified by the use of cigarettes to evaluate whether cigarette use may modify the association between alcohol and pancreatic cancer. The difference between the smoking strata was evaluated by comparing the logistic regression model with a product term (alcohol × cigarette) to the model without the product term using the log-likelihood ratio test.

The association between *ADH1B* rs1229984 or *ALDH2* rs671 and pancreatic cancer was first performed separately using unconditional logistic regression, adjusted for age, sex, educational level, cigarette smoking, oral hygiene, vegetable intake, history of allergy, history of diabetes/glucose intolerance, and BMI. Further analysis was performed by combining *ADH1B* rs1229984 and *ALDH2* rs671 into four ethanol-metabolizing groups for evaluating the association with pancreatic cancer risk. These four groups were: group 1: Fast *ADH1B* (*2/*2) + normal *ALDH2* (*1/*1); group 2: Fast *ADH1B* (*2/*2) + slow/non-functional *ALDH2* (*1/*2 + *2/*2); group 3: Slow *ADH1B* (*1/*1 + *1/*2) + normal *ALDH2* (*1/*1); and group 4: Slow *ADH1B* (*1/*1 + *1/*2) + slow/non-functional *ALDH2* (*1/*2 + *2/*2).

To evaluate whether the association between alcohol and pancreatic cancer could be modified by the genotypes of *ADH1B* rs1229984 and *ALDH2* rs671, unconditional logistic regression was first performed stratified by *ADH1B* rs1229984 and *ALDH2* rs671 separately and then by the four ethanol-metabolizing groups. The significance of effect modification was evaluated by comparing the logistic regression model with a product term (alcohol × *ADH1B* rs1229984 or alcohol × *ALDH2* rs671 or alcohol × ethanol-metabolizing group) to the model without the product term using the log-likelihood ratio test.

## Supplementary Information

Supplementary Information.

## Data Availability

All data generated or analyzed during this study are included in this published article.
