# Supplementary material for: No association between alcohol consumption and pancreatic cancer even among individuals genetically susceptible to the carcinogenicity of alcohol
Source: Sci Rep. 2021 Jul 15;11:14567. doi: 10.1038/s41598-021-94111-w (PMC8282609; doi:10.1038/s41598-021-94111-w)
Supplement: Supplementary file 1 — Supplementary Information. [file 41598_2021_94111_MOESM1_ESM.doc]

**No association between alcohol consumption and pancreatic cancer even among individuals genetically susceptible to the carcinogenicity of alcohol**

Yan-Shen Shan^1,2,*^, Li-Tzong Chen^3,4,5,6,*^, Chih-Hsing Wu^7,8,*^, Yin-Fan Chang^7^, Chih-Ting Lee^7^, **Nai-Jung Chiang**^3,4^, Ying-Jui Chao^1^, Chia-Jui Yen^4^, **Hui-Jen Tsai**^3,4^, Hsin-En Huang^9^,Chia-Rung Tsai^3^, Ya-Ling Weng^3^, Han-Chien Yang^3^, Hui-Chin Liu^10^, Jeffrey S. Chang^3^

1. Department of Surgery, National Cheng Kung University Hospital, National Cheng Kung University, 138 Sheng Li Road, Tainan 70456, Taiwan.
2. Institute of Clinical Medicine, College of Medicine, National Cheng Kung University, Tainan, 138 Sheng Li Road, Tainan 70456, Taiwan.
3. National Institute of Cancer Research, National Health Research Institutes, 367, Sheng-Li Road, Tainan 70456, Taiwan.
4. Department of Internal Medicine, National Cheng Kung University Hospital, National Cheng Kung University, 138 Sheng Li Road, Tainan 70456, Taiwan.
5. Department of Internal Medicine, Kaohsiung Medical University Hospital, Kaohsiung Medical University, Ziyou 1st Road, Sanmin District, Kaohsiung 80756, Taiwan
6. Institute of Molecular Medicine, College of Medicine, National Cheng Kung University, 138 Sheng Li Road, Tainan 70456, Taiwan.
7. Department of Family Medicine, National Cheng Kung University Hospital, National Cheng Kung University, 138 Sheng Li Road, Tainan 70456, Taiwan.
8. Institute of Geriatrics, College of Medicine, National Cheng Kung University, 1 University Road, Tainan, 701, Taiwan
9. Department of Family Medicine, Mennonite Christian Hospital, 44 Min Chuan Road, Hualien 970, Taiwan
10. Department of Nursing, National Cheng Kung University Hospital, National Cheng Kung University, 138 Sheng Li Road, Tainan 70456, Taiwan.

*These authors contributed equally to this work

**Correspondence to:** Jeffrey S. Chang, MD, PhD, MPH

National Institute of Cancer Research,

National Health Research Institutes

1F No 367, Sheng-Li Road, Tainan 70456, Taiwan

E-mail: jeffreychang@nhri.org.tw

Tel: 886-6-208-3422 ext 65160; Fax: 886-6-208-3427

Supplementary Table 1. The association between lifestyle and clinical factors and pancreatic cancer risk

| **Characteristics** | **Cases**  **N=419**  **n (%)** | **Controls**  **N=963**  **n (%)** | **Analysis 1**  **OR (95% CI)**^a^ | **Analysis 2**  **OR (95% CI)**^b^ |
| --- | --- | --- | --- | --- |
| **Cigarette smoking (pack-years)** |  |  |  |  |
| 0 (never-smoker) | 255 (60.9) | 746 (77.5) | Reference | Reference |
| 0.1-10 | 21 (5.0) | 72 (7.5) | 0.88 (0.51-1.53) | 1.00 (0.56-0.78) |
| 10.1-20 | 25 (6.0) | 41 (4.3) | 1.89 (1.07-3.31) | 1.74 (0.96-3.16) |
| >20 | 110 (26.2) | 98 (10.2) | 2.22 (1.51-3.25) | 2.01 (1.34-3.03) |
| Unknown | 8 (1.9) | 6 (0.6) | -- | -- |
| **Oral hygiene score**^c^ |  |  |  |  |
| 0 (Good) | 49 (11.7) | 305 (31.7) | Reference | Reference |
| 1 | 99 (23.6) | 318 (33.0) | 1.61 (1.09-2.38) | 1.44 (0.96-2.16) |
| 2 | 186 (44.4) | 267 (27.7) | 3.08 (2.12-4.48) | 2.42 (1.63-3.60) |
| 3 (Poor) | 77 (18.4) | 62 (6.4) | 4.70 (2.90-7.61) | 2.75 (1.64-4.60) |
| Unknown | 8 (1.9) | 11 (1.1) | -- | -- |
| **Vegetable consumption** |  |  |  |  |
| 3 portions or less per week | 130 (31.0) | 148 (15.4) | Reference | Reference |
| > 3 portions per week | 289 (69.0) | 815 (84.6) | 0.38 (0.28-0.51) | 0.46 (0.34-0.63) |
| **Allergy** |  |  |  |  |
| No | 334 (79.7) | 606 (62.9) | Reference | Reference |
| Yes | 85 (20.3) | 355 (36.9) | 0.54 (0.41-0.72) | 0.55 (0.41-0.75) |
| Unknown | 0 (0.0) | 2 (0.2) | -- | -- |
| **Diabetes mellitus/glucose intolerance (DM/GI)** |  |  |  |  |
| No DM/GI | 306 (73.0) | 886 (92.0) | Reference | Reference |
| < 2 years | 27 (6.4) | 24 (2.5) | 2.79 (1.53-5.08) | 2.00 (1.06-3.77) |
| > 2 years | 85 (20.3) | 52 (5.4) | 3.68 (2.50-5.43) | 2.99 (1.98-4.50) |
| Unknown | 1 (0.2) | 1 (0.1) | -- | -- |
| **BMI 2 years ago**^d^ |  |  |  |  |
| <18.5 | 7 (1.7) | 49 (5.1) | 0.49 (0.21-1.14) | 0.51 (0.21-1.21) |
| 18.5-23.9 | 146 (34.8) | 488 (50.7) | Reference | Reference |
| 24-26.9 | 143 (34.1) | 263 (27.3) | 1.38 (1.03-1.86) | 1.31 (0.95-1.79) |
| 27 or more | 121 (28.9) | 162 (16.8) | 2.10 (1.53-2.90) | 1.75 (1.24-2.47) |
| Unknown | 2 (0.5) | 1 (0.1) | -- | -- |

Abbreviations: BMI: body mass index; CI: confidence interval; DM/GI: diabetes mellitus/glucose intolerance; OR: odds ratio

a. OR and 95% CI of each independent variable in Model 1 was calculated using unconditional logistic regression, adjusted for sex, age, and education

b. Model 2 included all of the independent variables in the same model with additional adjustment for sex, age, and education

c. Oral hygiene score = tooth brushing + use of dental floss + regular dental visit, with tooth brushing: ≧2 times per day=0, <2 times per day=1; use of dental floss: yes=0, no=1; and regular dental visit: yes=0, no=1.

d. BMI at two years before the pancreatic cancer diagnosis for the cases or before the interview date for the controls
